# Immunohistochemical detection of connexin36 in sympathetic preganglionic and somatic motoneurons in the adult rat

**DOI:** 10.1016/j.autneu.2007.12.004

**Published:** 2008-05-30

**Authors:** Nephtali Marina, David L. Becker, Michael P. Gilbey

**Affiliations:** a Department of Physiology, University College London, London, WC1 E6BT, UK; b Department of Anatomy and Developmental Biology, University College London, London, WC1E 6BT, UK

**Keywords:** Sympathetic, Motoneurons, Gap junctions, Spinal cord, Rhythmic

## Abstract

Gap junctional communication in the adult CNS plays an important role in the synchronization of neuronal activities. In vitro studies have shown evidence of electrotonic coupling through gap junctions between sympathetic preganglionic motoneurons and between somatic motoneurons in the neonatal and adult rat spinal cord. Electrotonic transmission of membrane oscillations might be an important mechanism for recruitment of neurons and result in the generation of rhythmic sympathetic and somato-motor activity at the population level. Gap junctions in the adult spinal cord are constituted principally by connexin36 (Cx36). However, the distribution of Cx36 in specific neuronal populations of the spinal cord is unknown. Here, we identify Cx36-like immunoreactivity in sympathetic preganglionic and somatic motoneurons in thoracic spinal cord segments of the adult rat. For this purpose, double immunostaining against Cx36 and choline acetyltransferase (ChAT) was performed on transverse sections (20 μm) taken from spinal segments T6–T8. Cx36 punctate immunostaining was detected in the majority of ChAT-immunoreactive (-ir) neurons from lamina VII [intermediolateral cell column (IML) and intercalated cell group (IC)], lamina X [central autonomic nucleus (CA)] and in ventral horn neurons from laminae VIII and IX. Cx36 puncta were distributed in the neuronal somata and along dendritic processes. The presence of Cx36 in ChAT-ir neurons is consistent with electrical coupling between sympathetic preganglionic motoneurons and between somatic motoneurons through gap junctions in the adult spinal cord.

## 1 Introduction

Gap junctions are specialized cell–cell contacts that provide direct intercellular communication. They often occur as plaques that can contain thousands of individual gap junction channels. Each channel is formed by the association of 12 connexin (Cx) proteins. Eleven of the 20 members connexin family have been identified so far in the mammalian central nervous system (Nagy et al., 2004), but only Cx36 is expressed almost exclusively by neural cells (Belluardo et al., 2000; Condorelli et al., 2000; Rash et al., 2000). Previous studies have shown that gap junctions play an important role in the synchronization of neuronal activity in various areas of the neonatal and adult rodent brain, including the cortex (Deans et al., 2001), hippocampus (Hormuzdi et al., 2001; Buhl et al., 2003), thalamic reticular nucleus (Landisman et al., 2002), inferior olive (Long et al., 2002; De Zeeuw et al., 2003; Leznik and Llinas, 2005) and olfactory bulb glomeruli (Christie et al., 2005). Such junctions enable the intercellular flow of electric current (electrotonic transmission) which can result in the generation of coordinated activity within populations of neurons. In the rat spinal cord, electrotonic and dye coupling have been shown to occur between somatic motoneurons (Gogan et al., 1977; Walton and Navarrete, 1991; Chang et al., 1999; Mentis et al., 2002) and between sympathetic preganglionic motoneurons (Logan et al., 1996; Nolan et al., 1999). It has been suggested that synchronization of electrical activity through gap junctions plays an important role in the generation of somatomotor and sympathetic motor rhythms induced by NMDA and/or 5-HT (Marina et al., 2006; Tresch and Kiehn, 2000). In the spinal cord of adult rats, gap junctions are composed mainly by Cx36 and are distributed in the white matter and the grey matter (Rash et al., 2000; Lee et al., 2005). However, the expression of Cx36 protein in specific neural populations of the spinal cord remains unknown. In this study we used immunofluorescence to identify Cx36-like immunoreactivity in ChAT-positive putative sympathetic preganglionic and somatic motoneurons within thoracic spinal segments.

## 2 Materials and methods

### 2.1 Tissue preparation

Experiments were performed in accordance with the UK Animals (Scientific Procedures) Act 1986 and associated guidelines. Six male Sprague–Dawley rats (210–250 g, bred in-house at UCL) were anaesthetized deeply with 20% urethane (1.6 g kg^−^ ^1^ i.p.) and perfused through the ascending aorta with 100 ml of 0.9% saline at room temperature, followed by 500 ml of 4% paraformaldehyde in 0.1 M phosphate buffered saline (PBS, pH 7.4) at 4 °C. The thoracic spinal cord and the brain stem were removed, postfixed for 1 h and cryoprotected in 10% sucrose in 0.1 M PBS overnight at 4 °C. Transverse slices (20 μm thick) from spinal segments T6–T8 and caudal medulla were collected with a freezing microtome and stored in cryo-protectant (30% ethylene glycol and 20% glycerol in 0.05 M PBS) at −20 °C.

Caudal medulla slices were taken to assay specificity of the Cx36 antibody and to determine optimal antibody concentration using the inferior olive nucleus (ION) as a positive control. Cx36-immunostaining was analyzed in spinal segments T6–T8 because previous studies have shown evidence of electrotonic coupling among sympathetic preganglionic motoneurons and expression of Cx36 mRNA in somatic motoneurons from thoracic spinal cord slices (Logan et al., 1996; Parenti et al., 2000).

### 2.2 Immunohistochemistry

All series of tissue slices from all animals (20–30 slices/animal) were processed together in the same assay to ensure uniformity of immunostaining. After slices were washed in 0.1 M PBS with 0.1% Triton X (PBS-T), non-specific binding was blocked with 10% normal donkey serum (Sigma-Aldrich) in PBS-T (blocking medium) for 60 min. Tissue was then incubated in Cx36 antibody (1:100 dilution in blocking medium) at 4 °C overnight. The antibody was obtained by immunizing rabbits with a synthetic peptide corresponding to amino acids LQNTETTSKETEPDC of the murine Cx36 protein. The peptide sequence was compared to protein sequence databases from the Basic Local Alignment Search Tool (BLAST 2.0, NCBI, National Institutes of Health, USA), and showed local alignments with murine Cx36 protein only. The specificity of the Cx36 antibody was shown in Western blots and in preabsorption control experiments (Bittman et al., 2002). The slices were then washed again with PBS-T and incubated with fluorescent secondary antibody (Alexa Fluor 488 donkey anti-rabbit IgG, Molecular Probes, 1:500 dilution in blocking medium) for 60 min at room temperature, and washed again with PBS.

Choline acetyltransferase (ChAT) immunodetection was employed to identify putative sympathetic preganglionic and somatic motoneurons (Marina et al., 2002). Following staining for Cx36, slices were incubated in goat anti-ChAT affinity purified polyclonal antibody (Chemicon, 1:100 dilution in 1% normal donkey serum) overnight at 4 °C. Tissue was washed and incubated with Alexa Fluor 568 donkey anti-goat IgG antibody (Molecular Probes, 1:500 dilution in 1% normal donkey serum) for 60 min at room temperature, washed again in PBS and cover-slipped with Vectashield hard set mounting medium with the fluorescent nuclear dye DAPI (Agar Scientific, Stansted, Essex).

### 2.3 Image analysis

The anatomical distribution of Cx36 immunostaining was determined by visual inspection through 63× and 100× objectives with an Olympus BH2-RFCA epifluorescence microscope equipped with light filters (exciter 380–490 nm, emitter 519 nm for the green channel). ChAT-Immunoreactive (ir) neurons were considered positive for Cx36 if punctate staining was observed within the cell body or along the dendritic processes.

The cellular distribution of Cx36 in sympathetic preganglionic and somatic motoneurons was analyzed using a Leica TCS NT SP laser scanning confocal microscope with argon, krypton, and helium-neon lasers. Green and red channels were each scanned sequentially through a 63× objective. Alexa Fluor 488 labelling was observed by exciting at 488 nm and collecting at 515–550 nm. Alexa Fluor 568 labelling was visualized by using excitation at 568 nm and collecting at 590–626 nm. Serial optical sections were taken at intervals of 0.5 μm starting at the upper cell surface, i.e., the first optical section where cytoplasmic ChAT staining became visible, and all the way down through the cell to the lower cell surface, i.e., where ChAT staining was no longer detected. Composite figures were created for each optical section by merging both channels using Image J 1.37c (National Institutes of Health, USA). Colocalization of Cx36 (green) and cytoplasmic ChAT immunofluorescence (red) appeared as yellow. Thus, green puncta on the cell surface, i.e., surrounding ChAT-ir cytoplasm, suggested the presence of Cx36 on the plasma membrane; yellow intracellular puncta in ChAT-positive regions were indicative of cytoplasmic Cx36. Serial sections were projected as a single image to create a 2D reconstruction of 3D data set from the sections of the cell bodies and dendritic processes, using Leica Confocal Software (Leica Microsystems Heidelberg GmbH). Both the individual and projected images were saved directly to a computer as TIFF files. Images were minimally adjusted for brightness and contrast with Adobe Photoshop® 6 running on a Dell Pentium 4 PC. Images were then imported into Adobe illustrator® CS2 where groups of images were assembled and labelled.

## 3 Results

### 3.1 Connexin 36 immunostaining in the inferior olivary nucleus

**Fig. 1:**
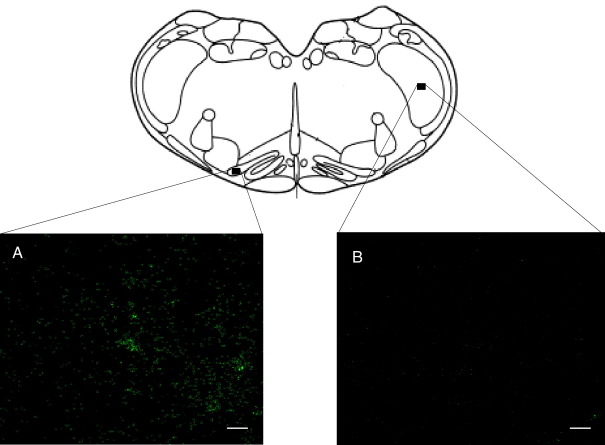
Cx36-immunoreactivity (ir) in a caudal medulla slice of the adult rat. Abundant fluorescent punctate staining was distributed in restricted regions within the inferior olivary nucleus (A). Punctate staining was almost undetectable in the trigeminal spinal nucleus (B). Confocal image is 2.5 μm thick (5 stacked optical sections taken at intervals of 0.5 μm scanned through a 63× objective). Scale bar = 10 μm.

Caudal medulla slices were immunostained for Cx36 to be used as a positive control. Cx36-immunostaining could only be detected using high magnification lenses (at least 63×) and appeared as minuscule round immunofluorescent puncta distributed in restricted regions within the inferior olivary nucleus (Fig. 1A). Cx36 expression in other medullary nuclei was extremely low, as illustrated in Fig. 1B, where Cx36-like-ir puncta are virtually undetectable in the trigeminal spinal nucleus. No staining was observed in negative controls where the primary antibody was omitted (data not shown).

### 3.2 Connexin36 immunostaining in the spinal cord

**Fig. 2:**
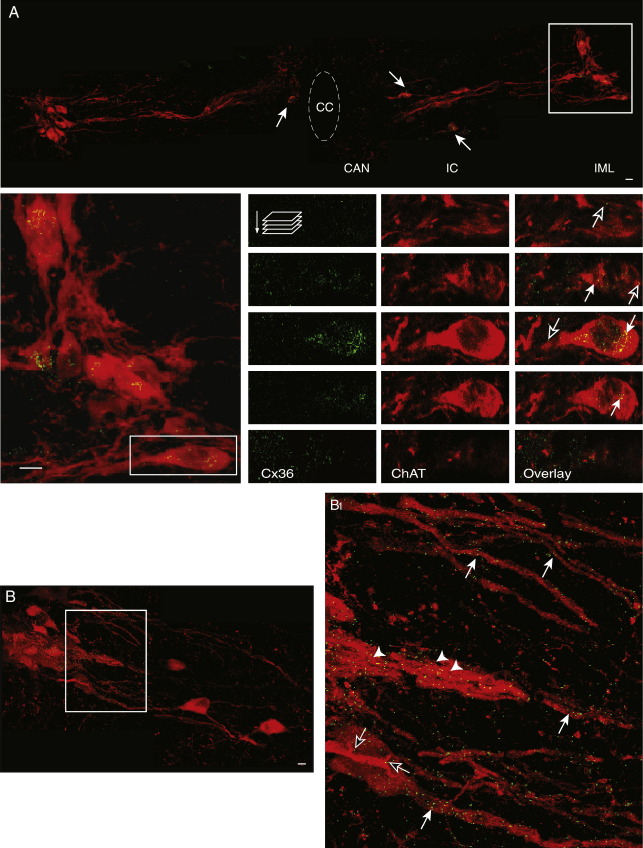
Representative examples of somal and dendritic labelling of Cx36 in sympathetic preganglionic motoneurons. Figures were assembled using overlayed confocal images (16 stacked optical sections per color taken at intervals of 0.5 μm) scanned through a 63× objective. A) Double immunnodetection of Cx36 (green) and ChAT (red) in a spinal cord slice from segment T8 showing the spatial arrangement of sympathetic nuclei: IML = intermediolateral cell column; IC = intercalated cell group; CAN = central autonomic nucleus; CC = central canal. Cytoplasmic co-localization of Cx36 and ChAT appears as yellow. Filled arrows show co-localization of Cx36 and ChAT in neurons located in CAN and IC. A1) Left, the area highlighted in A has been enlarged to show in more detail Cx36-like-ir puncta in sympathetic preganglionic motoneurons from the IML. Right, single optical sections taken at different depths of neuron highlighted in left. Membrane labelling was characterized by scarce green puncta on the cell surface, i.e., surrounding ChAT-ir cytoplasm (empty arrow on first, second and third optical sections); Cytoplasmic labelling was characterized by abundant yellow puncta located perinuclearly within the cell body (filled arrows in the second, third and fourth optical sections). B) Example of dendritic labelling of Cx36 in sympathetic preganglionic motoneurons from the IML. Figure was taken from spinal segment T6. B1) Dendritic labelling was characterized by fine punctate labelling distributed along the medially projecting primary dendrites, and thin filaments (filled arrows). Abundant punctate staining is observed in bundled dendrites (arrowheads). Empty arrows show green puncta between a fine fibre and a nearby cell body. Scale bar = 10μm.

Under low magnification (40× or less), Cx36 immunostaining is very hard to detect as immunofluorescent puncta are small and sparsely distributed in the ventral horn (Rash et al., 2000; Lee et al., 2005). Under high magnification (63× and higher), several Cx36-like-ir puncta were revealed within the soma of spinal neurons (Fig. 2).

### 3.3 Choline acetyltransferase immunostaining in the spinal cord

**Fig. 3:**
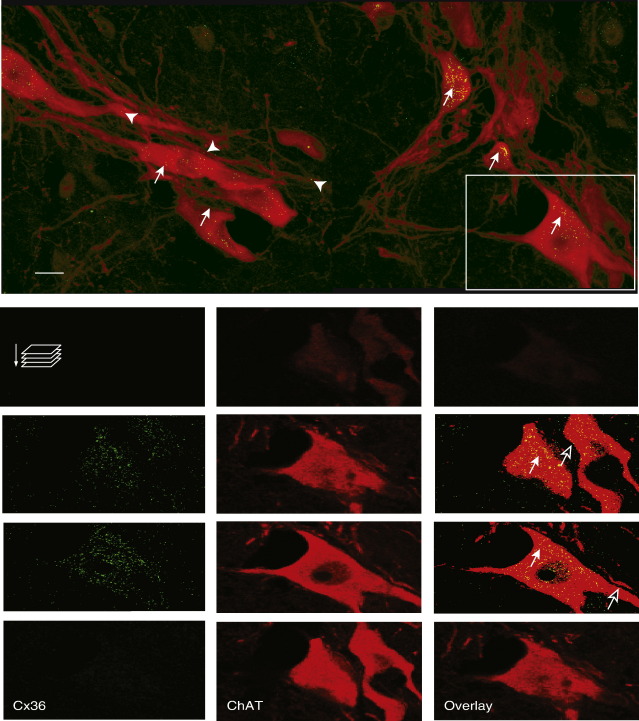
Example of somal labelling of Cx36 in somatic motoneurons. Immunohistochemical detection of Cx36 (green) in ChAT-ir neurons (red) from lamina IX. Overlapping colors are shown as yellow. The image was taken from the same slice as Fig. 2A. Top, Confocal image is 8 μm thick (16 stacked optical sections per color taken at intervals of 0.5 μm) scanned through a 63× objective. Filled arrows show colocalization of Cx36-like-ir puncta in the cytoplasm of ChAT-ir neurons. Dendritic labelling was characterized by scarce punctate labelling along the primary dendrites and thin filaments on somato-dendritic sites of close apposition (arrowheads). Bottom, single optical sections taken at different depths of neuron highlighted in top. Membrane labelling was characterized by scarce green puncta on the cell surface, i.e., surrounding ChAT-ir cytoplasm (empty arrow on second and third optical sections); Cytoplasmic labelling was characterized by abundant yellow puncta located perinuclearly within the cell body (filled arrows in the second and third optical sections). Scale bar = 10 μm.

Sympathetic preganglionic and somatic motoneurons were identified by immunohistochemical staining of ChAT in transverse spinal cord slices from thoracic spinal segments T6–T8. Intracellular ChAT immunostaining (red immunofluorescence) was detected extranuclearly in the cytoplasm and in the dendritic projections (Figs. 2 and 3). ChAT-ir neurons located in lamina VII (IML and IC) and lamina X (CA) were considered as sympathetic preganglionic motoneurons, according to the spatial arrangement described by Cabot (1990; see Fig. 2A). Lateral and medial ChAT-ir projections were easily identified in all sympathetic preganglionic motoneurons within the IML; dorsal and ventral dendritic projections were not identified in transverse spinal slices stained for ChAT. On the other hand, ChAT-ir neurons and their respective dendritic projections located in laminae VIII and IX were considered as somatic motoneurons, according to the anatomical boundaries described by Paxinos and Watson (1998; see Fig. 3).

### 3.4 Connexin 36 immunostaining in sympathetic preganglionic motoneurons

The presence of Cx36-like-ir puncta in transverse spinal cord slices taken from T6–T8 spinal segments immunostained for ChAT was analyzed with wide field epifluorescence microscopy. The cellular distribution of Cx36-like-ir puncta in putative sympathetic preganglionic and somatic motoneurons was analyzed in individual optical sections scanned sequentially along the cell body (z series) under high magnification. Optical sections were stacked to show a three-dimensional reconstruction of the neurons. Figs. 2 and 3 show examples of somal and dendritic labelling in ChAT-ir neurons taken from a representative spinal slice from animal number 5. Co-expression of Cx36 (green) and ChAT (red) immunofluorescence appears as yellow. Cx36 immunostaining in ChAT-ir neurons was similar in all the slices taken from all six animals used in this study. All ChAT-ir neurons located in the different sympathetic nuclei, i.e., IML, IC and CA, showed Cx36-like-ir puncta (See Fig. 2A and A1). Both, somal and dendritic puncta were observed in putative sympathetic preganglionic motoneurons. A detailed analysis of each optical section revealed that membrane labelling was very scarce; this was indicated by the small amount of green puncta surrounding ChAT-ir cytoplasm (Figs. 2A1). In contrast, moderate intracellular punctate staining was detected in ChAT-ir regions, i.e., perinuclearly, where the Cx36 is generated in the Golgi-ER complex, suggesting a substantial cytoplasmic production of Cx36 protein. On the other hand, Cx36 staining was consistently detected in ChAT-ir dendrites projected from sympathetic preganglionic neurons within the IML, i.e., lateral and medial projections. Dendritic labelling was characterized by fine puncta distributed along the primary dendrites and thin filaments. Robust Cx36-like staining was often observed in medially projecting bundled dendrites (Fig. 2B1). In some cases, fine puncta were also detected in areas where fine dendrites were seen in close proximity to a cell body (Fig. 2B1). As mentioned before, dorsal dendritic projections were not detected in transverse spinal slices stained for ChAT, and therefore, the presence of Cx36-like-ir puncta in dorsal dendrites could not be determined.

### 3.5 Connexin 36 immunostaining in somatic motoneurons

Cx36-immunoreactive puncta were consistently observed in somatic motoneurons from laminae VIII and IX. Cx36 labelling in somatic motoneurons was characterized by moderate punctate staining distributed perinuclearly in the cytoplasm and to a lesser extent on the plasma membrane. Punctate staining was also distributed along the dendritic arbour and it was often observed in dendro-dendritic and somato-dendritic sites of close apposition (Fig. 3).

## 4 Discussion

### 4.1 Methodological considerations

In the present study, connexin 36-immunoreactivity was identified in putative sympathetic preganglionic motoneurons and somatic motoneurons from the lower thoracic spinal cord of adult rats. The staining pattern obtained was similar to that described previously in the spinal cord and in the ION (Rash et al., 2000; Placantonakis et al., 2006). Even though the peptide used to immunize the rabbits showed local alignments with murine Cx36 protein only, it is important to mention that the specificity of the antibody has only been partially tested in preabsorption control experiments and in Western blots (Bittman et al., 2002). Further experimental evidence is required to fully determine the specificity of this antibody using tissue from Cx36 knock-out animals.

It is important to consider that connexin expression can only be estimated at the subcellular level using high magnification objectives. Cx 36-ir puncta are quite small, with an average diameter of 0.61 μ (Placantonakis et al., 2006) and Cx36 protein levels in the adult spinal cord are distinctly low. Therefore, visualization at low magnification can be extremely difficult; this point can also be illustrated by the apparent difference in the expression of Cx36mRNA levels in the adult spinal cord described in previous studies. Showing a panoramic photograph of the spinal cord at low magnification (judged by the scale bars), Lee et al. (2005) showed that Cx36 mRNA is strong in the neonatal rat spinal cord but in the adult spinal cord, expression is very low and barely above background. In contrast, Cx36 mRNA expression in the adult rat spinal cord can be clearly seen in higher magnification images by Chang et al. (2000). In the present study, it was also difficult to identify Cx36-like-ir puncta in panoramic images of the spinal cord (see Fig. 1A). However, a closer inspection revealed the presence of Cx36 within ChAT-ir neurons.

### 4.2 Cellular distribution of Cx36 immunostaining in ChAT-ir neurons

This study shows that ChAT-ir neurons from the adult rat thoracic spinal cord express the neuron-specific gap junction protein Cx36. Such labelling was detected both in the IML, IC and CA and in laminae VIII and IX and it was distributed in neuronal somata and along dendritic processes. Somal labelling was mainly characterized by moderate cytoplasmic punctate staining with scarce labelling on the plasma membrane and cell nucleus. Like all membrane proteins, connexins are synthesized by ribosomes and are transported in small vesicles that follow the cell's secretory pathway from the endoplasmic reticulum to the plasma membrane (Yeager et al., 1998; Martin and Evans, 2004). Removal of gap junctions occurs through the formation of annular gap junctions by phagocytosis and lysosomal degradation (Yeager et al., 1998). Therefore, the cytoplasmic labelling observed in putative sympathetic preganglionic motoneurons and in somatic motoneurons may represent the trafficking pathways of connexins leading to the formation and/or degradation of gap junction channels. Our results therefore support the contention that Cx36 is actively synthesized by the adult spinal cord. This is the first documentation of connexin 36-like immunostaining in ChAT-ir (putative sympathetic preganglionic) neurons in sympathetic nuclei of the spinal cord. Furthermore, our results are consistent with several studies, which have demonstrated that Cx36 mRNA is widely expressed in somatic motoneurons during the embryonic and neonatal life, and continues to be expressed in the adulthood (Chang et al., 1999; Chang et al., 2000; Parenti et al., 2000).

Cx36 labelling was scarce on the somatic plasma membrane of ChAT-ir neurons and it was never found in sites of close apposition between neighbouring cell bodies. However, abundant Cx36 labelling was identified in and along dendritic processes. In fact, Cx36-like-ir puncta were commonly detected in tightly bundled dendrites and in areas where fine dendrites were in close proximity with other cell bodies. The presence of Cx36 in these areas might represent a potential site for gap junctional coupling between dendrites and between the somata and the dendrites of the neighbouring sympathetic preganglionic and somatic motoneurons. This is consistent with in vitro studies using slice preparations, which have identified putative sites of coupling between dendrites and/or cell bodies in dye-coupled sympathetic preganglionic and somatic motoneurons (Becker and Navarrete, 1990; Shen and Dun, 1990; Mentis et al., 2002). This has also been confirmed at the ultrastructural level, where dendro-dendritic gap junctions have been identified in somatic motoneurons (van der Want et al., 1998). In fact, gap junctions in somatic motoneurons are distributed predominantly in dendro-dendritic and dendro-somatic sites and to a much lesser extent, in somato-somatic points (Matsumoto et al., 1988). Further ultrastructural studies are required to determine whether dendritic Cx36 protein is involved in the formation of homotypic gap junctional plaques among sympathetic preganglionic motoneurons or even heterotypic gap junctions between sympathetic preganglionic motoneurons and interneurons or astrocytes. For instance, previous studies have shown the presence of heterotypic gap junctions in the lumbar spinal cord, composed of Cx32 in superficial dorsal horn neurons on one side, and Cx43 in astrocytic processes on the other (Qin et al., 2005). Interestingly, neuronal tracing studies have proposed the presence of dendro-dendritic connections (presumably through gap junctions) between lamina V neurons from the dorsal horn and sympathetic preganglionic motoneurons from the IML (Cabot et al., 1994). It will be interesting to determine whether Cx36 in sympathetic preganglionic motoneurons is involved in the formation of heterotypic gap junctions with lamina V neurons.

### 4.3 Physiological role of gap junctional communication between sympathetic preganglionic motoneurons

Paired cell recordings have shown evidence of synchronization of action potential firing and subthreshold membrane potential oscillations between electrotonically coupled sympathetic preganglionic motoneurons (Logan et al., 1996; Nolan et al., 1999). Since action potential discharge in one of the sympathetic preganglionic motoneurons was almost simultaneously registered as a spikelet in the second sympathetic preganglionic neuron, it was considered that gap junctions mediating electrotonic coupling are located near the site of action potential generation (Logan et al., 1996). These data are consistent with the abundance of the gap junction protein Cx36 that we found between primary dendrites in sympathetic preganglionic motoneurons and supports the idea that electrical synapses might be located in dendro-dendritic sites of close apposition. Electrical synapses in sympathetic preganglionic motoneurons behave in a similar way to a low-pass filter, allowing electrotonic transmission of sub- and suprathreshold activity (Nolan et al., 1999). Therefore, it has been recently proposed that by increasing the degree of synchrony of discharges in the population of sympathetic preganglionic motoneurons, gap junctional communication might play a significant role in the generation of relatively low frequency coherent sympathetic motor rhythms induced by 5-HT in the adult rat (Marina et al., 2006).

### 4.4 Physiological role of gap junctional communication between somatic motoneurons

Several studies have shown evidence of electrical and dye coupling between somatic motoneurons in the neonatal spinal cord (Gogan et al., 1977; Becker and Navarrete, 1990; Walton and Navarrete, 1991; Chang et al., 1999; Mentis et al., 2002). Experiments using gap junction blockers and NMDA receptor antagonists have shown that coherent somato-motor rhythms result from the interaction between membrane properties at the single cell level and the transmission of this activity through gap junctions (see Kiehn and Tresch, 2002). It has been suggested that gap junction coupling is a very important factor that contributes to the synchronization of individual somatic motoneurons to produce a coordinated rhythmic motor output.

The dye transfer properties between neonatal somatic motoneurons change as development progresses, moving from Lucifer yellow to neurobiotin coupling (Becker and Navarrete 1990; Mentis et al., 2002). The number and intensity of labelling of dye-coupled somatic motoneurons are substantially reduced and becomes almost absent shortly after birth (Gogan et al., 1977; Becker and Navarrete, 1990; Walton and Navarrete, 1991; Chang et al., 1999; Mentis et al., 2002). Electrotonic coupling between somatic motoneurons in adult animals has only been observed in pathological conditions such as nerve injury (Chang et al., 1999). However, Cx36 mRNA expression in axotomized somatic motoneurons remains unaffected (Chang et al., 1999). In a similar way, recent evidence has shown that spinal cord injury is associated with a strong up-regulation of Cx43 in astrocytes, but in contrast, neural Cx36 expression remains unaffected (Lee et al., 2005). This suggests that an increased synthesis of Cx36 protein is not involved in the reappearance of coupling in adult injured animals (Chang et al., 1999). Therefore, it might be possible that the reappearance of coupling between adult somatic motoneurons following injury might be produced by different mechanisms, such as the activation of gap junctions that contain different connexins other than Cx36 or by the release of factors that affect either connexin assembly into functional gap junctions, or the open state of the channels. In fact, several studies have shown that gap junction coupling can be modified by the activation of multiple G protein-coupled receptors, by pH and intracellular calcium levels (see Bennett, 1997; Bennett and Zukin 2004). For instance, early postnatal blockade of NMDA receptors has been shown to prevent the decrease in electrotonic and dye coupling observed during the first postnatal week (Mentis et al., 2002). These data show the importance of gap junction coupling regulation by neuromodulators and suggest the possibility that electrotonic coupling between somatic motoneurons could also reappear in the adult animal under different kinds of pathological states, or even in physiological conditions. To date, this possibility has not been explored.

## 5 Conclusions

Our results confirm and expand previous observations regarding Cx36 expression in the spinal cord (Rash et al., 2000; Lee et al., 2005) and provide anatomical evidence that suggests that gap junctions composed by Cx36 may mediate electrotonic coupling between sympathetic preganglionic motoneurons and between somatic motoneurons. Further work using different approaches, such as blockers for Cx36 gap junction channels, Cx36-knock-out animals, transfection of sympathetic preganglionic motoneurons and somatic motoneurons with dominant-negative constructs of Cx36 and Cx36-specific antisense oligodeoxynucleotides, will reveal the precise role of Cx36 in the generation of coherent sympathetic and somato-motor rhythmic activity in the spinal cord of adult animals.
